# Reproducibility of the Pleth Variability Index in premature infants

**DOI:** 10.1007/s10877-017-0058-3

**Published:** 2017-08-31

**Authors:** Wilhelmina J. den Boogert, Hugo A. van Elteren, Tom G. Goos, Irwin K. M. Reiss, Rogier C. J. de Jonge, Victor J. van den Berg

**Affiliations:** 1 Division of Neonatology, Department of Pediatrics, Erasmus MC-Sophia Children’s Hospital, Room Sp-3434, P.O. Box 2060, 3000 CB Rotterdam, The Netherlands; 2 Department of Biomechanical Engineering, Faculty of Mechanical, Maritime and Materials Engineering, Delft University of Technology, Delft, The Netherlands

**Keywords:** Microcirculation, Premature neonates, Pleth Variability Index, Reproducibility

## Abstract

The aim was to assess the reproducibility of the Pleth Variability Index (PVI), developed for non-invasive monitoring of peripheral perfusion, in preterm neonates below 32 weeks of gestational age. Three PVI measurements were consecutively performed in stable, comfortable preterm neonates in the first 48 h of life. On each occasion, pulse oximeter sensors were attached to two different limbs for 5 min. Reproducibility was assessed with the intra-class correlation coefficient (ICC) and Bland–Altman analysis. A total of 25 preterm neonates were included. Inter-limb comparison showed fair to moderate ICC’s with 95%-confidence intervals (95%-CI). Left hand–right hand ICC = 0.498, 95%-CI (0.119–0.753); right foot–right hand ICC = 0.314 (−0.088–0.644); right foot–left foot ICC = 0.315 (−0.089–0.628). Intra-limb comparison showed fair to moderate ICC for right foot–right foot ICC = 0.380 (−0.014–0.677); and good ICC for right hand–right hand ICC = 0.646 (0.194–0.852). Bland–Altman plots showed moderate reproducibility of measurements between different limbs and of the same limb in consecutive time periods, with large biases and wide limits of agreement. The findings from this study indicate that PVI measurement is poorly reproducible when measured on different limbs and on the same limb in stable and comfortable preterm neonates.

## Introduction

The principal goal of fluid administration is to increase cardiac output without the accumulation of fluid, causing tissue edema. For this reason, a predictive index of fluid responsiveness would be useful. Some studies have demonstrated that the Pleth Variability Index (PVI) is reliable to predict fluid responsiveness in ventilated adults in the operating room and the ICU [7, 9, 10, 15, 22]. The PVI is a parameter based on the changes in the perfusion index (PI) during a complete respiratory cycle [8]. It can be measured continuously by most Masimo pulse oximeters at the bedside and is calculated based on the difference between the lowest and highest PI (PVI = ((PI_max_ − PI_min_)/PI_max_) × 100%) [15]. The PI is calculated by indexing the infrared pulsatile signal (AC) from the blood flow in the arterioles against the non-pulsatile signal (DC) absorbed by skin, other tissues, and non-pulsatile blood, and is expressed as a percentage (PI = (AC/DC) × 100%) [15].

Others, however, have shown that PVI is not suitable to predict fluid responsiveness in critically ill, hemodynamically unstable adult patients receiving norepinephrine [16]. Due to physiological differences between newborns, children and adults—such as in chest, lung and arterial compliance [1, 5]—the predictive ability of PVI cannot be extrapolated directly from adults to infants or (preterm) neonates. Studies evaluating the effectiveness of PVI to predict fluid responsiveness in children reported contradictory results. Three studies in mechanical ventilated children found a significant difference in PVI between responders and non-responders based on an increase in stroke volume index (SVI) [6, 11, 19]. Yet another study in mechanical ventilated children showed no predictive relation between PVI and fluid responsiveness [17]. All above-mentioned studies investigated the usefulness of PVI to predict fluid responsiveness in children beyond neonatal age. One pilot study showed that PVI might be a useful indicator of volume-response hypotension in newborn infants during surgery [2].

Since preterm infants regularly are administered fluids as treatment for hypotension, PVI is of interest for monitoring their fluid management in the neonatal intensive care unit (NICU) setting. Accumulation of fluid in tissue would cause tissue edema, in particular in the lungs, and would influence closure of the ductus arteriosus and necessitate prolonged mechanical ventilation [3]. If PVI is found to be able to predict fluid responsiveness, it could be a valuable non-invasive device in this population. However, as neonates are completely different to adults in both physiology and behavior, it is important to study the feasibility of a new diagnostic tool in this population.

Therefore, the aim of this study was to assess the reproducibility of the PVI measurement on the same limb and on two different limbs in preterm infants younger than 32 weeks of gestational age (GA).

## Methods

The study protocol was approved by the medical ethical review board of the Erasmus MC and written informed consent was given by all parents. Newborns between 26 and 32 weeks of gestational age, younger than 48 h after birth and admitted to the NICU of the Erasmus MC - Sophia Children’s hospital were eligible for this study. Patients with any known cardiac or chromosomal defect were excluded from this study. As the largest study on PVI in newborns was performed in spontaneously breathing newborns and the majority of the patients in the NICU is spontaneously breathing, we decided to include mechanical ventilated as well as spontaneously breathing preterm infants. In order to create a most heterogeneous group, to achieve the highest external validity, no further exclusion criteria were applied.

### Study procedures

To calculate the PVI, two Masimo Radical seven pulse oximeters (Masimo Corp., Irvine, CA, USA) with NeoPt Softtouch sensors were connected to a laptop. Three 5-min measurements were performed. On each occasion, two different limbs were each fitted with a pulse oximeter sensor. The first measurement compared right wrist vs. left wrist. The second measurement compared right wrist versus right foot. The third measurement compared right foot vs. left foot. If a sensor could not be attached to the wrist because a peripheral intravenous or arterial catheter was in place, it was attached to the palm of the hand. For all study subjects, sensors were placed by the same operator. Recording of data started when a clear PVI signal was obtained within 5 min after the sensors were placed. PVI values were recorded with a frequency of one per second for a total of 5 min, resulting in 300 data points per measurement. Every 124–145 s a status report from the Masimo was received, resulting in the loss of nine data point. Since “0” is not an actual PVI value but a representation of a poor signal, or poor calculation of PVI, all data points with a value of “0” were discarded. The mean of the PVI values per measurement was calculated over the remaining values.

Pulse oximeter derived variables such as heart rate, oxygen saturation and PI were synchronously recorded. Since PVI is influenced by behavioral status, all measurements were conducted while the neonates were quiet and comfortable. Furthermore, they were left undisturbed during the measurements. Changes in behavioral or circulatory status were reported [14].

The following baseline characteristics were retrieved: gestational age (GA), birth weight, sex, mode of delivery, type of ventilation, age at start of measurement, and Apgar scores.

### Statistical analysis

After confirming our data had a normal distribution, differences in means of the PVI inter- and intra-limb were assessed with paired *t* tests. Reproducibility of the mean PVI per sensor side was assessed with two-way mixed intra-class correlation coefficients (ICC) [20]. This coefficient can vary between 0 and 1.0, with 1.0 reflecting perfect agreement and 0 no agreement between the two measurements. Reproducibility is considered very good at an ICC > 0.80; good at 0.61–0.80; fair to moderate at 0.20–0.60; and poor if below 0.20 [18]. For further analysis of the reproducibility Bland–Altman plots with bias and 95% limits of agreement were created by calculating the differences between the means of measurements [4].

To assess if the average PVI of 300 PVI values per measurement influences the reproducibility of the PVI, Bland–Altman analyses of single PVI values for one measurement per patient (left foot–right foot) were made.

Continuous data are presented as median and range for non-normally distributed variables and as mean and standard deviation (SD) for normally distributed parameters. Non-continuous variables are presented as number of events and percentages of total.

Statistical analysis was performed using SPSS21 (IBM Corp., Armonk, New York) and Prism 5 (GraphPad Software Inc., La Jolla, CA, USA).

## Results

A total of 25 preterm newborns were included between March 2014 and August 2014. Their background characteristics are presented in Table 1. Two infants were intubated and received synchronized intermitted mandatory ventilation (SIMV) during data collection; 21 infants required nasal continuous positive airway pressure (CPAP) or nasal intermittent positive pressure ventilation (NIPPV); and two infants received respiratory support from a nasal cannula without positive end expiratory pressure (PEEP).

**Table 1:** Background characteristics of the 25 included preterm neonates

Male sex^a^	9 (36%)
Gestation age (weeks)^b^	30 0/7 (27 2/7–31 5/7)
Birth weight (g)^b^	1175 (430–1910)
Apgar score 1 min^b^	6 (2–9)
Apgar score 5 min^b^	8 (1–10)
Twins^a^	10 (40%)
Caesarian section^a^	17 (68%)
Age at start measurement (hours)^c^	32 (10)
Type of ventilation	
SIMV^a^	2 (8%)
CPAP or NIPPV^a^	21 (84%)
Nasal cannula without PEEP^a^	2 (8%)

^a^Number of subjects (%)

^b^Median (range)

^c^Mean (SD)

### Observations

In total 72 measurements were performed in 25 children. For 21 neonates all three measurements were successful. Three measurements could not be performed due to an arterial line or peripheral catheter at the intended site of the sensor. During six individual measurements the neonate was described as restless and during 66 measurements the neonates were quiet or a sleep. The manufacturer of the Masimo sensors describes that PVI will be calculated after 2 min if a clear plethysmographic waveform is displayed. However, in the majority of cases it took over 5 min to calculate PVI at the start of the measurement. During 12 measurements, (in nine neonates; 36% of all patients), PVI was not calculated for a brief period, i.e. a mean of 85 s, with a maximum of 163 s. During these periods no changes in behavioral status were seen and PI was presented for the whole period.

### Reproducibility

Eighteen hundred PVI data points were recorded for every patient. For each sensor side the average PVI during 300 s was calculated. In total five comparisons were made to determine reproducibility of the PVI. First, three inter-limb comparisons: left hand (LH)–right hand (RH), right hand (RH)–right foot (RF) and right foot (RF)–left foot (LF). Second, to determine a difference over time on the same limb, two intra-limb comparisons were made: RH–RH and RF–RF. Paired *t* tests showed significant differences for two of the comparisons, i.e. inter-limb RF–RH (RF = 19.3, RH = 27.7, p < 0.001) and intra-limb RH–RH (RH1 = 22.9, RH2 = 27.7, p = 0.002).

Inter-limb comparison showed fair to moderate ICC for LH–RH, RF–RH and RF–LF. Intra-limb comparison showed fair to moderate ICC for RF–RH and good ICC for RH–RH 0.646. Table 2 gives an overview of the paired *t* test p value and ICC per comparison.

**Table 2:** Paired *t* tests and Intra class correlation coefficient inter limb and intra limb measurements

Measurement	Paired *t* test	ICC (95% confidence interval of the difference)
Inter-limb		
LH–RH (n = 22)	p = 0.234	0.498 (0.119–0.753)
RF–RH (n = 22)	p = 0.000	0.314 (−0.088 to 0.644)
RF–LF (n = 25)	p = 0.515	0.315 (−0.089 to 0.628)
Intra-limb		
RF–RF (n = 23)	p = 0.222	0.380 (−0.014 to 0.677)
RH–RH (n = 22)	p = 0.002	0.646 (0.194–0.852)

**Fig. 1:**
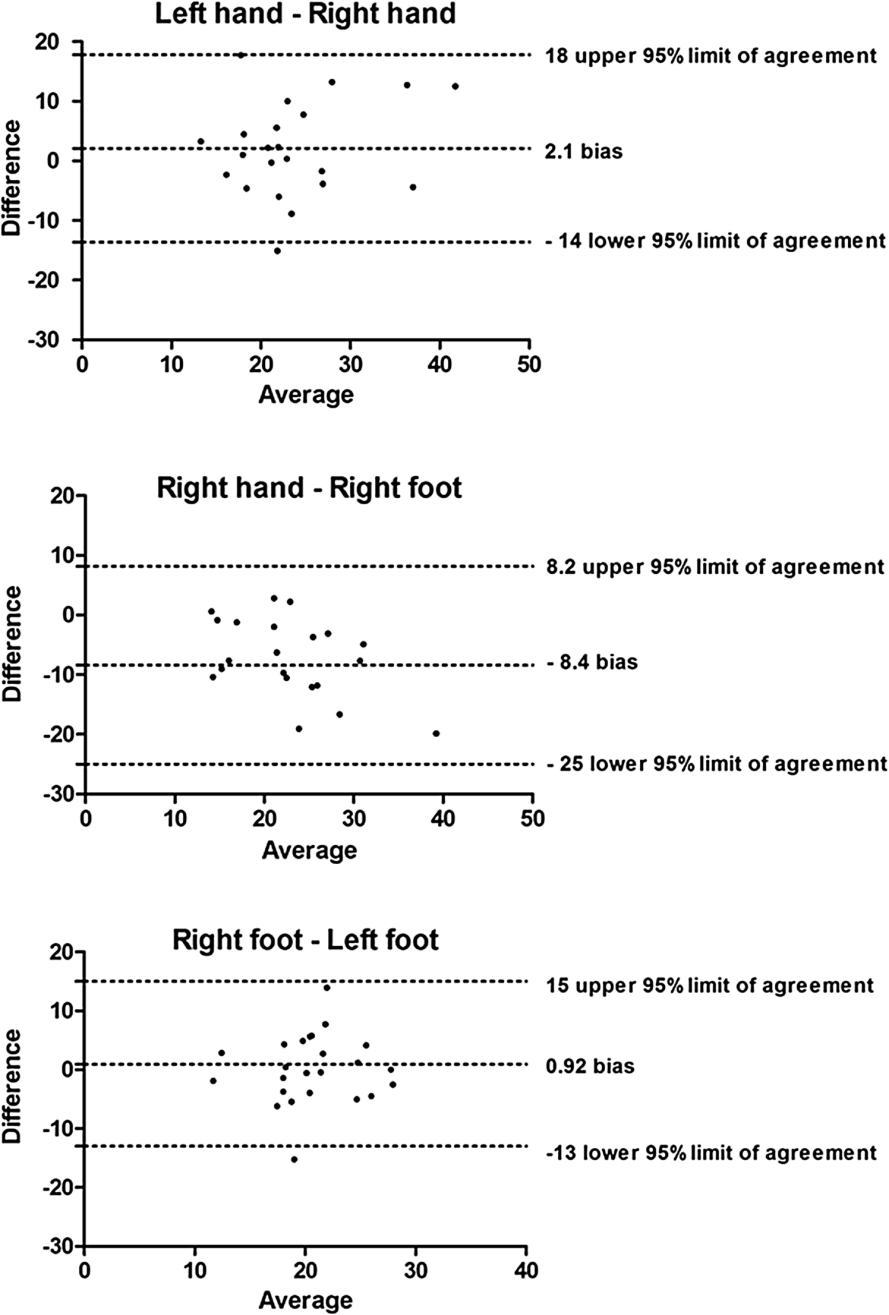
Bland–Altman plots showing the agreement of inter-limb measurments

**Fig. 2:**
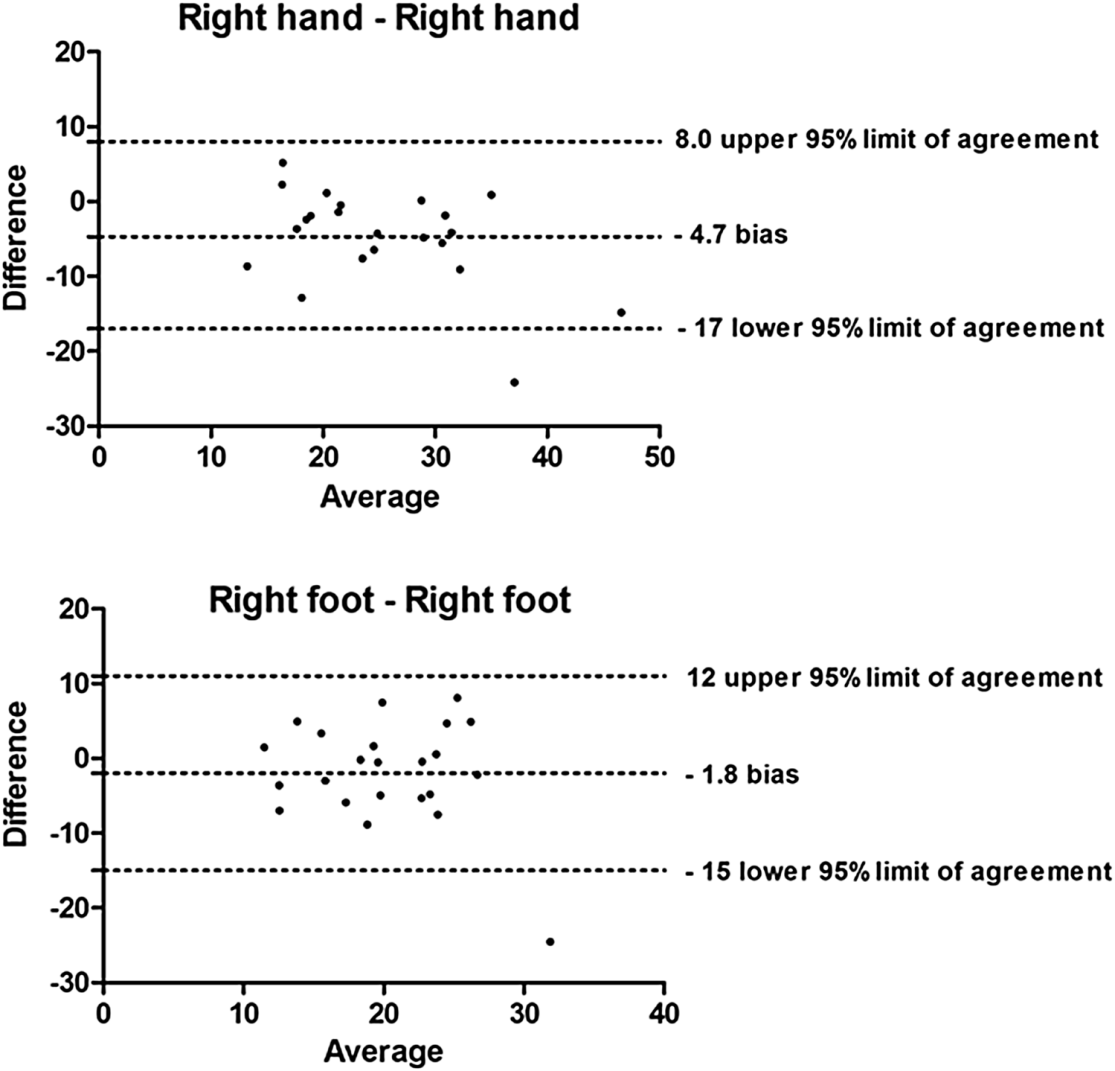
Bland–Altman plots showing the agreement of intra-limb measurements

To visualize the reproducibility of PVI and to assess bias between the measurements, Bland–Altman plots and 95% limits of agreement were created. Figure 1 shows Bland–Altman plots of inter-limb measurements; Fig. 2 shows Bland–Altman plots of intra-limb measurements.

For one type of measurement (LH–RF), Bland–Altman plots for all 25 patients were created to assess if there was a difference with the average PVI value and to assess if there is a bias by calculating the average PVI of the 300 values per measurement (Table 3). Only six Bland–Altman plots of the feet had a bias lower than 1.5 and almost all measurements had a wide spread of 95% limits of agreement.

**Table 3:** Agreement of PVI-measurement comparing left foot–right foot in individual patients

Patient no.	N (amount of seconds with PVI result)	Bias (PVI value)	95% Limits of agreement (PVI value)
1	266	−5.1	−12–1.5
2	257	7.7	4.7–11
3	258	4.9	−2.2–12
4	266	−20	−29 to −11
5	260	−5.4	−8.9 to −1.9
6	253	2.8	−1.7–7.3
7	189	2.8	−0.19–5.7
8	282	0.43	−4.8–5.7
9	264	4.3	−3.8–12
10	255	−0.02	−3.1–3.1
11	249	−2.7	−19–13
12	184	14	14–14
13	271	−15	−31–0.29
14	261	−4.8	−14–4.8
15	249	−3.8	−20–12
16	258	1.3	−4.1–6.8
17	246	−5.9	−13–1.1
18	256	−2.9	−7.1–3.2
19	259	5.8	−0.27–12
20	260	−4.4	−8.1 to −0.77
21	264	−0.40	−3.2–2.4
22	254	−0.35	−4.0–3.3
23	257	5.4	−1.1–12
24	258	−1.1	−4.4–2.2
25	243	−3.7	−5.7–1.7

Results of Bland–Altman analysis

## Discussion

To the best of our knowledge, this is the first reproducibility study of PVI in preterm neonates. The reproducibility of PVI was found to be fair to moderate during the first 48 h of life of these circulatory stable preterm neonates between 26 and 32 weeks of GA. For two comparisons (RF–RH and RH–RH) a paired t-test showed significant differences between measurements. One intra-limb comparison RH–RH showed good reproducibility. For all other comparisons, an ICC between 0.31 and 0.60 (fair to moderate) was found with wide 95% confidence intervals. The Bland–Altman analysis showed a poor reproducibility of the PVI with large biases and wide 95%-limits of agreement. The Bland–Altman analysis of single PVI values of one measurement did not show better results. The heterogeneity in background characteristics of the patients increases the external validity of our study results.

PVI reproducibility in neonates has not been studied before, although three studies of PVI measurements in neonates have been published [2, 14, 21]. Latini et al. established reference values for 242 spontaneously breathing term neonates; the median PVI value was 20 (19–20) [14]. Vidal et al. included 56 newborns below 29 weeks of gestational age and established a median PVI value of 22 (18–27) [21]. Bagci et al. showed in a pilot study that PVI might be a useful indicator of volume-response hypotension in newborn infants during surgery [2]. It is unfortunate that these studies did not indicate whether the reported PVI values are a mean of several data points or just a value on one point [14, 21]. The mean PVI values per measurement of our study were in the same range as found by both studies [14, 21]. However, in the study of Vidal et al. PVI was measured on either foot, whereas we did not find reproducibility of PVI on the foot.

Studies in adults noted that PVI could predict fluid responsiveness in mechanically ventilated hemodynamic stable patients in the operating theatre [8, 15, 22]. PVI could be of interest for monitoring fluid management in the NICU setting for critically ill neonates or in the operating theatre. Before we can evaluate the usefulness of this parameter for the prediction of fluid responsiveness, we first wanted to demonstrate whether this parameter would be reproducible in general, therefore this study was designed to validate the PVI in circulatory stable, spontaneously breathing preterm neonates.

Besides the fact that PVI was not found to be reproducible we found that even though other parameters were stable and behavioral status did not change, our results are consistent with a wide range of PVI values measured in 300 s.

Our study showed that in the majority of the cases it took more than 5 min before PVI was calculated by the Masimo pulse oximeter, and in nine (36%) neonates the PVI was not calculated for a mean of 85 s, with a maximum of 163 s. Since all other parameters (heart rate, oxygen saturation and PI) showed uninterrupted data, and a normal plethysmographic waveform was displayed, we cannot explain this observation. Latini et al. showed that PVI was significantly influenced by the behavioral status [14]. We hypothesized that a change in behavioral status could cause the loss of PVI, but behavioral status did not change during the short periods when PVI was not calculated.

Kinoshita et al. evaluated the reproducibility of PI in 30 preterm infants younger than 32 weeks of gestational age. They concluded that PI was highly reproducible (ICC 0.982) on the same limb and between limbs [13]. Since the calculation of the PVI is primarily based on the PI we expected that PVI would be reproducible in preterm neonates. To compare our results with those found by Kinoshita et al. and to understand why we failed to show good reproducibility in PVI measurement, we performed post-hoc analysis of the PI as well. Similar to Kinoshita et al. reproducibility of PI on the same limb was found to be good (RH–RH ICC 0.768 (95% CI 0.504–0.893) and RF–RF ICC 0.842 (95% CI 0.661–0.930)) and Bland–Altman plots showed very small biases and 95% limits of agreement. Taking the formula into account (PVI = ((PI_max_ − PI_min_)/PI_max_) × 100%), it is difficult to explain why PVI in this study is not reproducible on the same limb even when PI is reproducible. An explanation of this is to be sought in the (time) algorithm of the PI calculation which is not accessible to the researchers. Also, the high respiratory rate of preterm infants compared to adults might be of influence for the PVI calculation.

Another explanation for our results could be due to a physiological difference of the peripheral perfusion and on the sensor site. The newborns included to the study were younger than 48 h (mean 32 h). Therefore, it is possible that a hemodynamically significant patent ductus arteriosus may affect the reproducibility of PVI between different limbs. The ductus arteriosus causes ductal and arterial shunts which may influence both ventricular preload and cardiac output. Since the PVI is influenced by the perfusion index, which differs pre and post ductal [12], the difference between pre and post ductal sensor sides could explain that the PVI in not reproducible between limbs in preterm neonates. Most of previous studies of PVI were performed in the operation rooms. There are several physiological differences of PVI measurements between the operating room and neonatal care, which should be taken in consideration. Furthermore the conditions in the operation room are different in comparison with the neonatal care. During surgery motion artifacts are eliminated, there is autonomic suppression under anesthesia and there is a lack of positive pressure ventilation due to consistent volumes [8].

Several limitations of this study should be addressed. The sample size of this study was relatively small with a total of 25 patients. However, both the poor ICCs and the wide spread in the Bland–Altman plots suggest that larger samples will not necessarily demonstrate better reproducibility. A second limitation is that we could not measure at the exact same place and time. However, this is the reflection of clinical practice and since behavioral and circulatory states of the patient (heart rate and oxygen saturation) did not change in 5 min, the hypothesis was that the PVI would be similar during these measurements. A final limitation is that PVI values are compared as a mean of 300 values. When the average of 300 values is calculated a lot of information will be lost. Still, Bland–Altman plots of single PVI values of one measurement LH–RH RH–RH per patient showed that the majority of the measurements had a bias >1.5 and wide spread of 95% limits of agreement and therefore were not reproducible as well.

In conclusion, literature in adults showed that PVI could be of interest for monitoring fluid registration in the NICU setting. However, this parameter should be validated in preterm neonates first. This study demonstrates that measurement of the PVI is not reproducible in stable and comfortable, but spontaneously breathing preterm neonates and seems therefore not feasible as a diagnostic parameter in neonatal care. But, to explore the usability in non-spontaneously breathing neonates, a reproducibility study must be performed in this specific population.

## Abbreviations

CI: Confidence interval
CPAP: Continuous positive airway pressure
CVP: Central venous pressure
GA: Gestational age
ICC: Intra-class correlation coefficient
NICU: Neonatal intensive care unit
NIPPV: Nasal intermittent pressure ventilation
PDA: Patent ductus arteriosus
PEEP: Positive end expiratory pressure
PI: Perfusion index
PP: Pulse pressure
PPV: Pulse pressure variation
PVI: Pleth Variability Index
SIMV: Synchronized intermitted mandatory ventilation
SD: Standard deviation
SVI: Stroke volume index
